# Spatiotemporal Dynamics and Assembly Mechanisms of Bacterial Communities in Tropical-Subtropical Coastal Waters of the Leizhou Peninsula, China

**DOI:** 10.3390/microorganisms14061359

**Published:** 2026-06-17

**Authors:** Junyu Wei, Menghan Gao, Yingyi Fan, Sen Ai, Mi Zhang, Yulei Zhang, Huaming Wu, Zhangxi Hu

**Affiliations:** Guangdong Provincial Key Laboratory of Aquatic Animal, Disease Control and Healthy Culture, Laboratory of Marine Ecology and Aquaculture Environment of Zhanjiang, College of Fisheries, Guangdong Ocean University, Zhanjiang 524088, China; arctichakimifish@gmail.com (J.W.); gaomenghan0914@163.com (M.G.); fanyingyi1@stu.gdou.edu.cn (Y.F.); aisennn@foxmail.com (S.A.); zhang_mi05@126.com (M.Z.); zhangyl@gdou.edu.cn (Y.Z.); huamingwu@gdou.edu.cn (H.W.)

**Keywords:** bacterial community, spatiotemporal distribution, community assembly, co-occurrence networks, Leizhou Peninsula

## Abstract

Bacterial communities play vital roles in coastal biogeochemical cycling and ecological stability. Despite their importance, a significant knowledge gap exists regarding their spatiotemporal dynamics and assembly mechanisms in the tropical coastal waters of the Leizhou Peninsula, China. To investigate the bacterial community structure, co-occurrence networks, and assembly processes, we conducted 16S rRNA gene amplicon sequencing on water samples collected seasonally from August 2022 to June 2023. The bacterial communities were dominated by *Proteobacteria* and *Cyanobacteria*, and were characterized by a distinct warm-season peak in the relative of *Cyanobium*. Alpha diversity indices exhibited significant seasonal fluctuations, reaching a minimum in August (autumn) and a maximum in December (winter). These variations were strongly regulated by water temperature and phosphate availability. Redundancy analysis (RDA) identified salinity as the primary deterministic factor shaping community composition. Seasonal environmental heterogeneity, rather than spatial variation, primarily governed bacterial community dynamics. We also observed a seasonal succession in community assembly mechanisms with deterministic filtering dominated in autumn, whereas stochastic processes prevailed in other seasons. Predicted functional profiles indicated a stable core metabolism, although local anthropogenic inputs stimulated specific metabolic adaptations in industrial and aquaculture zones. Our findings reveal that seasonal environmental filtering (especially temperature and salinity) and a shifting balance between stochastic and deterministic assembly processes govern bacterial dynamics in this tropical coastal ecosystem, with anthropogenic inputs modulating local metabolic functions. This study provides fundamental insights into the mechanisms maintaining microbial diversity and stability in tropical coastal waters facing seasonal and human pressures.

## 1. Introduction

Bacteria and other microorganisms play a fundamental role in aquatic ecosystems, where they underpin productivity, biogeochemical cycling, and ecological stability by driving organic matter transformation, nutrient turnover, and functional resilience [1,2,3,4,5]. They regulate aquatic and marine ecosystem function through spatially structured, environmentally selected communities [6,7], whose diversity and functional traits govern nutrient transformations, organic matter degradation [8,9], and system-level responses to environmental heterogeneity and disturbance [10]. Bacterial diversity, particularly in terms of richness and evenness [11,12], shapes both the efficiency of biogeochemical cycles and the ecological response to changing environmental conditions [13,14]. Therefore, characterizing bacterial community composition and diversity is essential for understanding aquatic ecosystems and informing strategies to maintain ecosystem health.

In recent decades, high-throughput sequencing technologies, including long-read methods [15], have revolutionized microbial ecology in aquatic ecosystems by enabling detailed spatiotemporal characterization of bacterial community dynamics and their ecological drivers [16,17,18]. For instance, Gilbert et al. [19] identified day length as the primary factor explaining the seasonal succession patterns of microbial communities in the English Channel. Zhu et al. [20] demonstrated that salinity and dissolved inorganic nitrogen significantly drove the spatial distribution of bacterioplankton and algal–bacterial co-occurrence networks in the Pearl River Estuary. Isabwe et al. [21] observed that dispersal limitation was the key ecological process governing microbial community assembly in river–reservoir systems. Furthermore, Ye et al. [22] found that soil microbial networks in subtropical coastal wetlands exhibited significantly higher complexity in winter. Collectively, these studies exemplify how high-throughput sequencing has deepened our understanding of the complex interplay between microbial communities and environment factors. However, despite these extensive global and regional investigations, systematic research focusing on the annual cross-seasonal dynamics of bacterial communities specifically in the Leizhou Peninsula remains scarce.

The Leizhou Peninsula, one of the three largest peninsulas in China, is located at the southernmost tip of the mainland, covering an area of approximately 8500 km^2^. Its highly indented coastline features numerous bays and islands, with the peninsula bordered by the South China Sea to the east, the Beibu Gulf to the west, and the Qiongzhou Strait to the south. This topography creates a semi-enclosed coastal environment with a transition from nearshore estuarine waters to offshore open ocean [23,24]. In recent years, the coastal ecosystem of the Leizhou Peninsula has been increasingly threatened by intensive anthropogenic disturbances, characterized by elevated fluxes of land-sourced pollutants and the accumulation of hazardous heavy metals in coastal sediments [25,26]. Ecological studies in the coastal waters of the Leizhou Peninsula have increasingly focused on the spatiotemporal distribution of major nutrients, including nitrogen and phosphorus [27,28], heavy metal contamination in surface sediments [26], and chlorophyll *a* concentrations as a proxy for phytoplankton biomass [23,29]. Despite progress in related fields, only a limited number of studies have specifically explored the annual cross-seasonal dynamics of bacterial communities in this unique tropical coastal ecosystem, leaving a critical gap in our understanding of microbial drivers’ responses to environmental heterogeneity.

In this study, we focused on the coastal waters of the Leizhou Peninsula and systematically investigated the bacterial community structure and its spatiotemporal dynamics across four seasons. Through high-throughput sequencing and multivariate statistical analyses, we further explored the co-occurrence network patterns and community assembly mechanisms, as well as their coupling relationships with environmental drivers, to enhance our understanding of the microbial dynamics and ecological stability of this important tropical ecosystem. To our knowledge, this is the first study to systematically reveal the diversity and composition of bacterial communities across four seasons in the coastal waters of the Leizhou Peninsula, China. This work provides a scientific basis for understanding microbial community responses to environmental fluctuations and for developing conservation and management strategies for the tropical coastal ecosystem.

## 2. Materials and Methods

### 2.1. Sampling Sites, Sample Collection and Analyses

**Figure 1:**
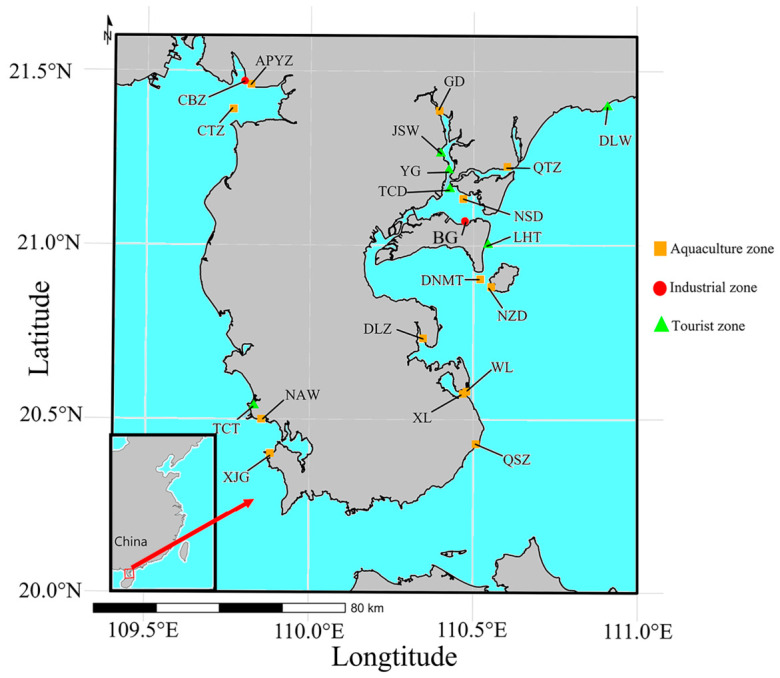
Location of the sampling sites in the Leizhou Peninsula, China.

The Leizhou Peninsula, located at the southernmost tip of mainland China, forms the southwestern region of Guangdong Province, China. It spans coordinates from 20.22° to 21.95° N and 109.67° to 110.97° E (Figure 1). Over the period from August 2022 to June 2023, four seasonal expeditions (autumn (August 2022), winter (December 2022), spring (March 2023), and summer (June 2023)) were conducted. A total of 21 sample sites were established along the peninsula’s coastline, covering different functional zones (Figure 1). Based on their predominant anthropogenic activities and land use characteristics, these sampling sites were grouped into three distinct categories: (1) aquaculture zones (*n* = 13), areas characterized by intensive mariculture activities, including fish, shrimp, and shellfish farming; (2) tourist areas (*n* = 6), coastal sites with high human visitation, encompassing beaches, resorts, and recreational waterfronts; and (3) industrial zones (*n* = 2), locations adjacent to major industrial complexes, including an iron and steel plant and a nuclear power plant. This classification was designed to evaluate how different human activities influence coastal microbial communities and biogeochemical processes across the peninsula. At each station, one water sample was collected per seasonal survey, and three technical replicates were processed during DNA extraction, PCR amplification, and sequencing, and the resulting sequences were merged prior to bioinformatic analysis to improve sequencing depth and reduce technical variability.

In situ environmental factor analyses and metabarcoding samples preparation were followed our previous work [30,31].

### 2.2. DNA Extraction, PCR Amplification, Data Processing, and Bioinformatic Analyses

Total genomic DNA was extracted from filtered seawater samples using the cetyltrimethylammonium bromide (CTAB) protocol provided by LC-Bio Technology Co., Ltd. (Hangzhou, China). A nuclease-free water blank was included as a negative control. The extracted DNA was eluted in 50 µL of elution buffer and stored at −80 °C prior to downstream analyses. The V3–V4 hypervariable regions of the bacterial 16S rRNA gene were amplified using the universal primers 341F (5′-CCTACGGGNGGCWGCAG-3′) and 805R (5′-GACTACHVGGGTATCTAATCC-3′) with sample-specific barcodes [32]. A polymerase chain reaction (PCR) was carried out in 25 µL reaction mixture containing 25 ng of template DNA, 12.5 µL of 2× Taq PCR StarMix (TransGen Biotech, Beijing, China), 2.5 µL of each primer (2 µM), and nuclease-free water to a final volume. Thermal cycling conditions consisted of an initial denaturation at 98 °C for 30 s, followed by 32 cycles of 98 °C for 10 s, 54 °C for 30 s, and 72 °C for 45 s, with a final extension at 72 °C for 10 min. Amplicons were visualized on 2% agarose gels, purified using AMPure XT magnetic beads (Beckman Coulter Genomics, Brea, CA, USA), and quantified with a Qubit 4.0 fluorometer (Invitrogen, Carlsbad, CA, USA). Library quality was verified using an Agilent 2100 Bioanalyzer (Agilent, Santa Clara, CA, USA), and equimolar pooling was performed before sequencing on the Illumina NovaSeq 6000 platform (2 × 250 bp paired-end) at LC-Bio Technology Co., Ltd. (Hangzhou, China).

Raw reads were demultiplexed based on unique barcodes, which were subsequently removed along with primer sequences. Paired-end reads were merged using Pear (v0.9.6), followed by quality filtering with fqtrim (v0.94). Chimeric sequences were identified and removed using Vsearch (v2.3.4). Amplicon sequence variants (ASVs) were generated using the DADA2 plugin in QIIME2 (v.2023.7) after denoising and length trimming; singletons were discarded. Taxonomic assignment was performed against the SILVA 138 database at a confidence threshold of 0.7.

### 2.3. Statistical Analyses

Statistical analyses were conducted using R software (v.4.4.3) and QIIME2. Alpha diversity indices (Shannon, Simpson, Chao1, richness, Pielou’s evenness, and ACE) and Good’s coverage were calculated in QIIME2. Seasonal differences were tested using the Kruskal–Wallis test followed by pairwise Wilcoxon rank-sum tests (independent samples) with Bonferroni correction. The adjusted significance threshold of *p* < 0.0083 corresponds to six pairwise comparisons among four seasons (0.05/6). Effect sizes were quantified using the rank biserial correlation coefficient (r). Beta diversity was assessed by non-metric multidimensional scaling (NMDS) based on Bray–Curtis and Jaccard distances using the “vegan” package. Spearman correlation analysis examined relationships between alpha diversity indices and environmental factors, visualized as heatmaps. Redundancy analysis (RDA) using the “vegan” package evaluated the influence of environmental factors on community composition. Co-occurrence networks were constructed via Spearman correlations and visualized in Gephi (v.0.10.1), with the calculation of topological parameters and identification of keystone taxa. To reduce network complexity and avoid spurious correlations, the Spearman rank correlations among ASVs were calculated and filtered for significance and reliability (*p* < 0.05, |r| > 0.6) using the “psych” package in R. The Sloan neutral model was fitted via nonlinear regression using the beta distribution, with ASVs classified as common (within 95% CI) or rare (outside CI). βNTI and RCbray were calculated based on 999 randomizations (tip-label shuffling for βNTI, richness-constrained randomization for RCbray), with thresholds of ±2 and ±0.95, respectively. The Stegen framework then categorized assembly processes as heterogeneous selection (βNTI > +2), homogeneous selection (βNTI < −2), dispersal limitation (|βNTI| ≤ 2 & RCbray > +0.95), homogenizing dispersal (|βNTI| ≤ 2 & RCbray < −0.95), and undominated (remainder). Functional profiles and heatmaps of functional abundances were generated with the “pheatmap” package.

## 3. Results

### 3.1. Composition and Distribution of Bacterial Communities in Coastal Waters of the Leizhou Peninsula

**Figure 2:**
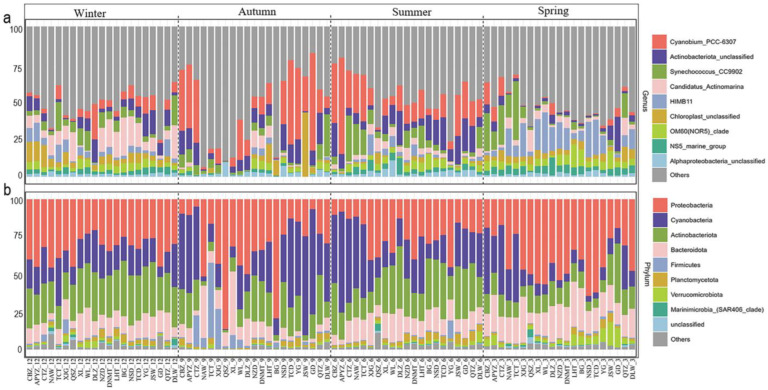
Characterization of bacterial communities in coastal waters of the Leizhou Peninsula, China ((**a**) genus level; (**b**) phylum level; other: bacterial unclassified).

A total of 7,060,159 raw sequences were obtained, yielding 41,012 ASVs after quality control and DADA2 denoising (Table S1). Taxonomic annotation revealed 78 phyla, 206 classes, 492 orders, 860 families, and 2129 genera. The relative abundance of the top 10 phyla and genera were shown in Figure 2. Less abundant microbial groups were grouped as “other”, accounting for 2.29% of sequences at the phylum level and 29.99% at the genus level.

The bacterial communities were dominated by *Proteobacteria* (32.4%), *Cyanobacteria* (23.3%), *Actinobacteriota* (20.6%), *Bacteroidota* (13.5%), and *Firmicutes* (2.54%), in decreasing order of relative abundance (Figure 2). *Proteobacteria* and *Cyanobacteria* were the top two dominant phyla. In terms of seasonal variation, *Proteobacteria* exhibited relative abundances of 40.72%, 24.44%, 31.36%, and 33.18% in spring, summer, autumn, and winter, respectively. However, *Cyanobacteria* showed abundances of 15.75%, 27.76%, 31.14%, and 18.54% across the same four seasons.

At the genus level, a substantial proportion of sequences (34.49%) could not be assigned to known genera. Among the classified sequences, the bacterial communities were dominated by *Cyanobium*_PCC-6307 (12.46%), *Synechococcus*_CC9902 (6.74%), *Candidatus*_*Actinomarina* (5.05%), and HIMB11 (4.99%) (Figure 2). *Cyanobium*_PCC-6307 was the dominant genus, with relative abundances ranging from 3.28% in spring to 21.97% in autumn, showing a distinct seasonal peak in autumn.

### 3.2. Diversity of Bacterial Communities in Coastal Waters of the Leizhou Peninsula, China

**Figure 3:**
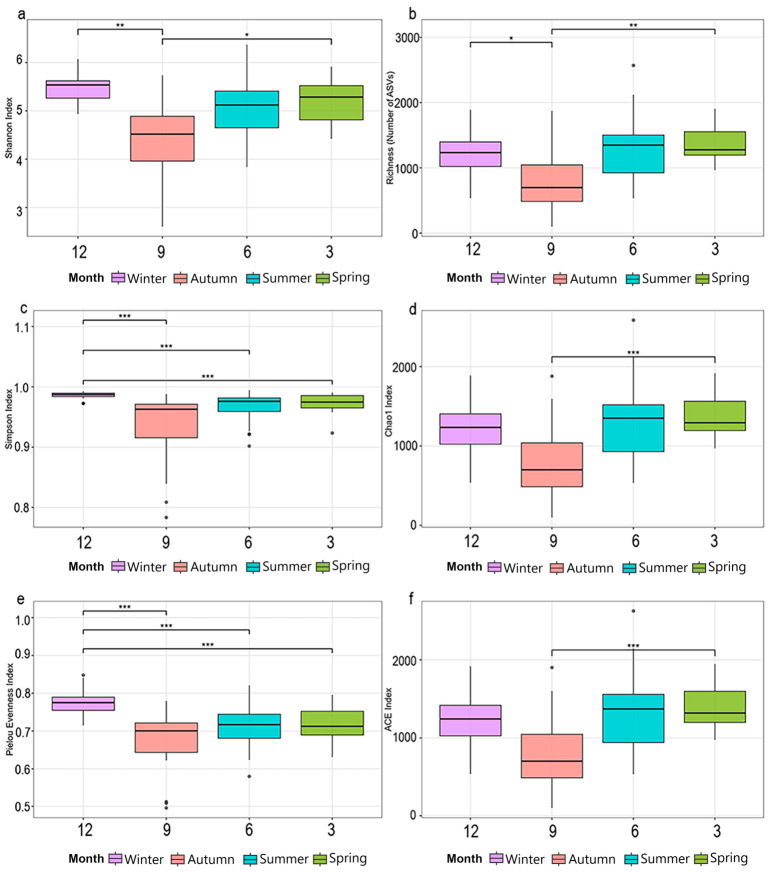
The α-diversity analysis of bacterial communities in coastal waters of the Leizhou Peninsula, China (Shannon (**a**), Richness (**b**), Simpson (**c**), Chao1 (**d**), Pielou (**e**), ACE (**f**); Pairwise comparisons were performed using Wilcoxon rank-sum tests with Bonferroni correction. Asterisks (*) indicate significant differences after Bonferroni correction (adjusted *p*-values): * adjusted *p* < 0.05, ** adjusted *p* < 0.01, *** adjusted *p* < 0.001.

The seasonal variations in alpha diversity indices across the four seasons were examined (Figure 3). The Kruskal–Wallis test revealed significant differences among seasons for the Shannon (*p* < 0.001), Simpson (*p* < 0.001), Chao1 (*p* < 0.001), Richness (*p* < 0.001), Pielou’s evenness (*p* < 0.001), and ACE (*p* < 0.001) indices. Pairwise Wilcoxon rank-sum tests with Bonferroni correction were used to adjust the significance threshold to *p* < 0.0083 to identify significant seasonal shifts. Effect size analysis revealed a large difference between December and September (r = 0.658), while other significant pairwise comparisons showed moderate effect sizes (r = 0.336–0.483), confirming the biological relevance of the observed seasonal differences in Shannon diversity. Specifically, significant differences were observed between spring and autumn for the Shannon, Chao1, Richness, and ACE indices. Additionally, significant differences were found between autumn and winter for the Shannon, Simpson, Richness, and Pielou’s evenness indices. Autumn consistently exhibited the lowest diversity, whereas winter showed the highest values for most indices (Figure 3a–f). Shannon indices ranged from 2.60 to 6.07 across all samples, with autumn recording the lowest mean value; other alpha diversity indices including Chao1, ACE, and Pielou’s evenness showed broadly consistent seasonal patterns. Good’s coverage exceeded 0.996 for all samples (average of 0.998), confirming that sequencing depth was sufficient to capture the majority of microbial taxa.

**Figure 4:**
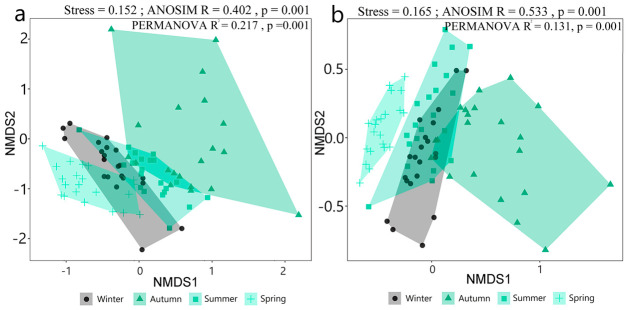
The β-diversity of bacterial communities in coastal waters of the Leizhou Peninsula, China (NMDS analysis using (**a**) Bray–Curtis and (**b**) Jaccard distances).

Non-metric multidimensional scaling (NMDS) based on Bray–Curtis (Figure 4a) and Jaccard distance matrices (Figure 4b) was used to further assess spatial and temporal similarities in bacterial community composition. NMDS stress values (< 0.2) indicated reliable ordination of community dissimilarities. ANOSIM confirmed significant seasonal differences in bacterial community composition for both distance measures (*p* < 0.01). To further validate these findings, PERMANOVA was additionally performed, confirming significant seasonal structuring of bacterial communities based on both Bray–Curtis (R^2^ = 0.217, *p* = 0.001) and Jaccard (R^2^ = 0.131, *p* = 0.001) distances, with season accounting for 21.7% and 13.1% of the total community variation, respectively.

**Figure 5:**
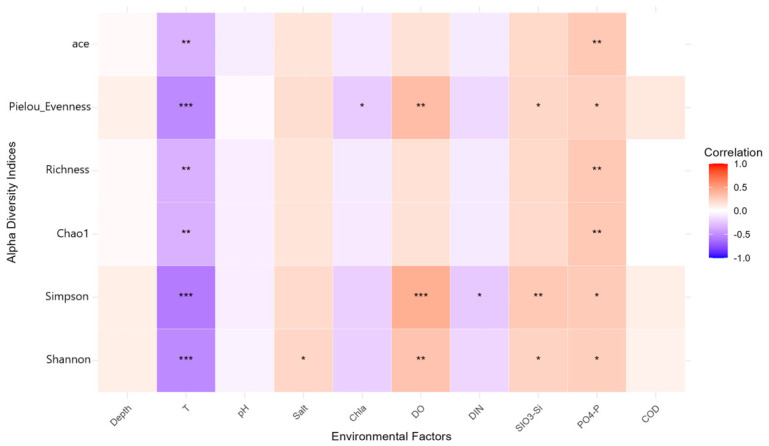
The correlation heatmap shows relationships between alpha diversity indices and environmental factors, with colors representing correlation coefficients. Red for positive and blue for negative, range from –1.0 to 1.0. Asterisks indicate significance levels: * *p* < 0.05, ** *p* < 0.01, *** *p* < 0.001.

Correlation analysis showed that water temperature was significantly negatively correlated with the Shannon (R = −0.510, *p* < 0.001), Simpson (R = −0.574, *p* < 0.001), and Pielou’s evenness (R = −0.513, *p* < 0.001) indices (Figure 5). In contrast, phosphate showed significant positive correlations with all α-diversity indices (R ranging from 0.250 to 0.288, *p* < 0.05 for Shannon, Simpson, and Pielou’s evenness; *p* < 0.01 for Chao1, Richness, and ACE), highlighting that both temperature and phosphate availability are key determinants of microbial alpha diversity across seasons.

### 3.3. Analysis of Factors Influencing Bacterial Community Differences

**Figure 6:**
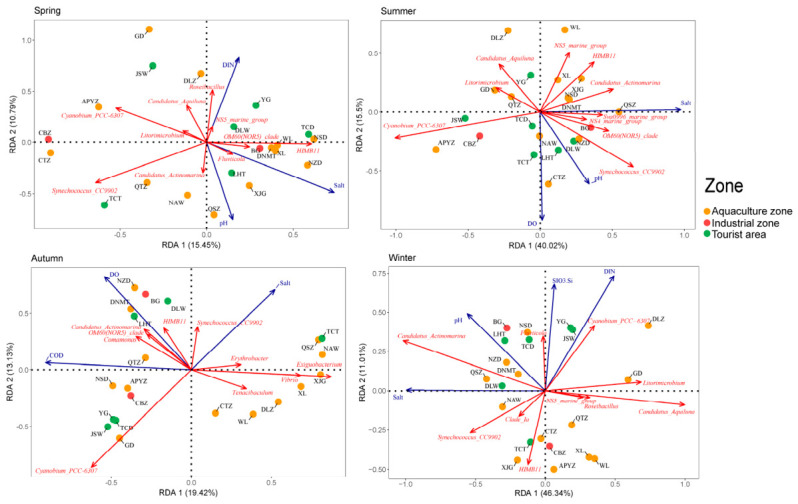
Redundancy analysis of bacterial communities in coastal waters of the Leizhou Peninsula, China. Based on Hellinger–transformed ASV abundances. Environmental variables were selected using stepwise ordination with 9999 permutations. Significance of individual environmental factors was assessed by permutation tests (9999 permutations, *p* < 0.05).

Redundancy analysis (RDA) was performed to assess the influence of environmental factors on bacterial community composition across seasons (Figure 6; Table 1). The results revealed pronounced seasonal shifts in the key drivers. Salinity emerged as a dominant and consistent factor, exhibiting the highest explanatory power in both summer (*p* < 0.001) and winter (*p* < 0.001), explaining 20.7–37.9% of bacterial community compositional variation across these seasons, as determined by single-variable RDA. Dissolved oxygen (DO) was also a recurrent influence, reaching significance in spring, summer, and autumn (*p* < 0.05). Furthermore, chemical oxygen demand (COD) and pH were identified as significant contributors in specific seasons, particularly in spring and autumn for COD (*p* < 0.05) and autumn and winter for pH (*p* < 0.05). Temperature and depth showed significant but more seasonally restricted effects, primarily in summer and winter (*p* < 0.05). Collectively, these findings underscore that salinity, DO, and COD are the paramount environmental factors shaping bacterial community dynamics in the coastal waters of the Leizhou Peninsula.

### 3.4. Characteristics of Bacterial Community Co-Occurrence Networks

**Figure 7:**
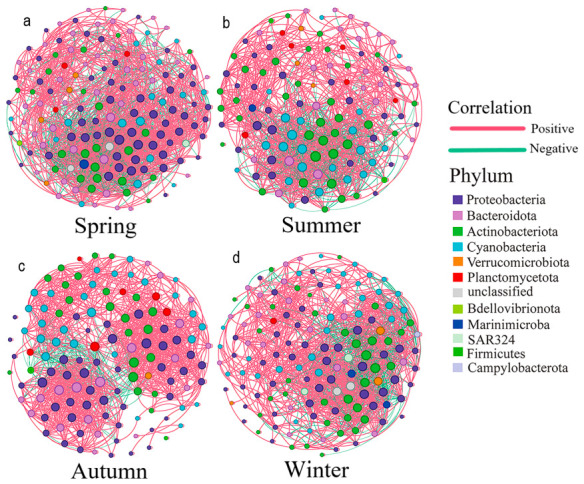
Bacterial community co-occurrence network analysis. (**a**–**d**) Network visualizations for each season. Each node represents a bacterial phylum. Node size is proportional to the relative abundance of that phylum in the corresponding season, and larger nodes indicate higher abundance. Networks were constructed based on Spearman correlations with FDR correction, filtered at |r| ≥ 0.6 and *p* < 0.05. Red edges indicate positive correlations; green edges indicate negative correlations.

Co-occurrence network analysis was performed on bacterial communities in coastal waters of the Leizhou Peninsula across four seasons (Figure 7). Results revealed that interspecies associations within bacterial communities were predominantly positive across all seasons. The spring network exhibited the highest node count (163) and edge count (2228), with the greatest average degree (27.337) and shortest average path length (2.321). This topology indicates a highly connected community in spring (Figure 7a). The summer network showed reduced size but maintained a high clustering coefficient (0.599) and density (0.171), reflecting a compact topology (Figure 7b). In autumn, the network structure shifted tremendously. While the average degree (22.078) remained comparable to other seasons, the modularity (0.437) reached its annual maximum (Figure 7c). By winter, the network became sparser, with lowest density (0.148) and reduced modularity (0.341) compared to autumn (Figure 7d).

**Figure 8:**
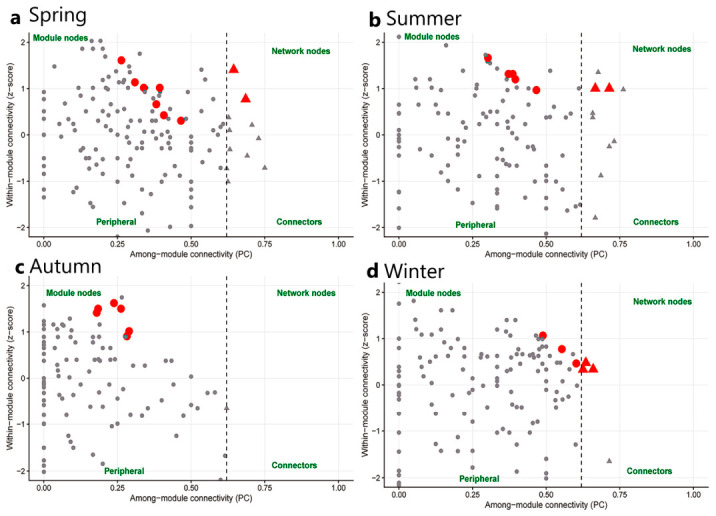
Bacterial community keystone species analysis. (**a**) Spring, (**b**) Summer, (**c**) Autumn, (**d**) Winter. Key species analysis based on within-module connectivity (z-score) and among-module connectivity. Red circles: hubs that are not connectors; red triangles: hubs that are also connectors; gray circles: non-hubs and non-connectors; gray triangles: connectors that are not hubs.

Typically, network nodes, module nodes, and connectors are considered keystone species in community construction, potentially playing crucial roles in maintaining community structure [33]. Removal of these taxa could lead to module and network collapse. In our study, bacterial communities in coastal waters of the Leizhou Peninsula across seasons lacked module nodes and showed no prominent network nodes in any season, with connectors present only sporadically: 16 in spring, 18 in summer, 2 in autumn, and 4 in winter (Figure 8a–d).

### 3.5. Analysis of Bacterial Community Assembly Processes

**Figure 9:**
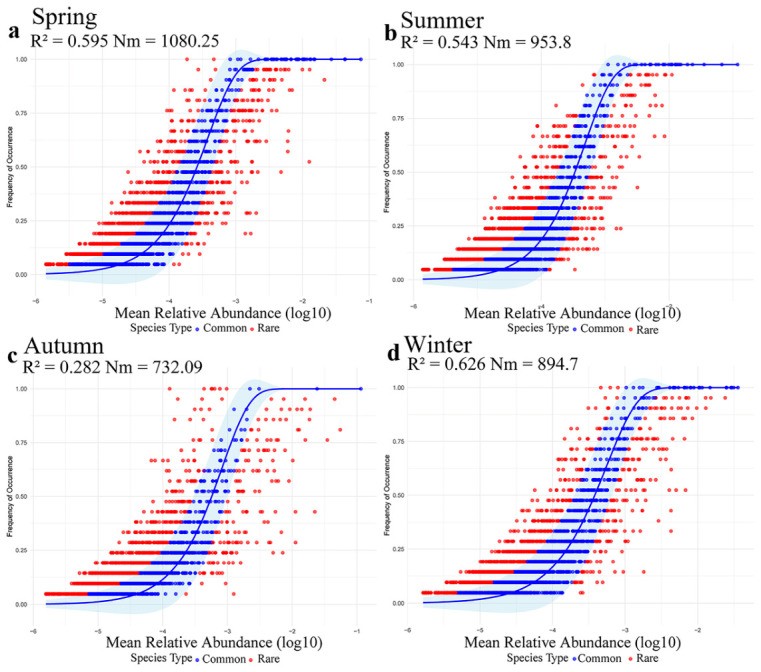
Inference of bacterial community assembly processes across seasons. Abundance–occurrence frequency relationships for spring (**a**), summer (**b**), autumn (**c**), and winter (**d**). Common species are shown in blue and rare species in red. The solid blue line represents the best-fit curve, while the dashed blue lines indicate the 95% confidence intervals. R^2^ values reflect the goodness of fit.

**Figure 10:**
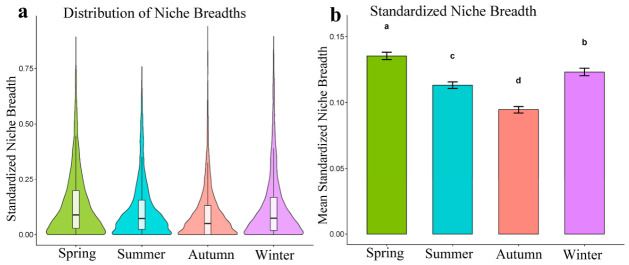
Inference of bacterial community assembly processes across seasons. (**a**) Violin plot of niche breadth distribution, illustrating the distribution characteristics of bacterial taxa niche breadth in each season. (**b**) Bar plot of niche breadth showing mean values across different seasons. Different letters (a–d) indicate statistically significant differences based on Kruskal–Wallis with Dunn’s test.

**Figure 11:**
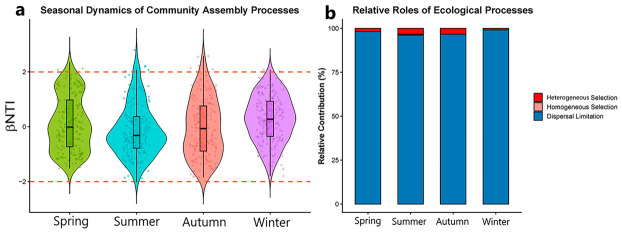
Inference of bacterial community assembly processes across seasons. (**a**) Violin plots showing the distribution of the β-Nearest Taxon Index (βNTI) within each season. Horizontal dashed lines at +2 and −2 denote the significance thresholds, where |βNTI| > 2 indicates deterministic processes and |βNTI| < 2 indicates stochastic processes. (**b**) Stacked bar charts illustrating the relative contributions of distinct ecological assembly processes quantified using the Stegen null-model framework based on βNTI.

To evaluate the relative contributions of stochastic and deterministic processes to seasonal bacterial community assembly, we employed the Sloan neutral community model and niche breadth analysis. The abundance–occurrence relationship analysis revealed the highest goodness of fit in winter (R^2^ = 0.626; Figure 9d), indicating that stochastic processes dominated community assembly in this season; in contrast, autumn exhibited the lowest goodness of fit (R^2^ = 0.282) (Figure 9c). Niche breadth distributions provided complementary insights (Figure 10a, b). The distribution of violin plots indicated that the coastal bacterial communities of the Leizhou Peninsula were rich in rare specialists, which formed the cornerstone of community diversity, but were also subject to strict environmental constraints (Figure 10a). Analysis of variance and post hoc tests (ANOVA, *p* < 0.05) showed that the niche widths of the four seasons exhibited completely independent and significant differences (Figure 10b). The βNTI distributions indicated that autumn exhibited the highest frequency of outlier values extending beyond the ±2 thresholds (Figure 11a). By partitioning these assembly processes using the Stegen framework, our stacked bar charts revealed that stochastic processes were overwhelmingly driven by dispersal limitation, which constituted the vast majority of community turnover across all seasons. Deterministic processes, including heterogeneous selection and homogeneous selection, accounted for a minor proportion of community assembly across all seasons (Figure 11b).

### 3.6. Predicted Metagenomic Functions of Bacterial Communities

**Figure 12:**
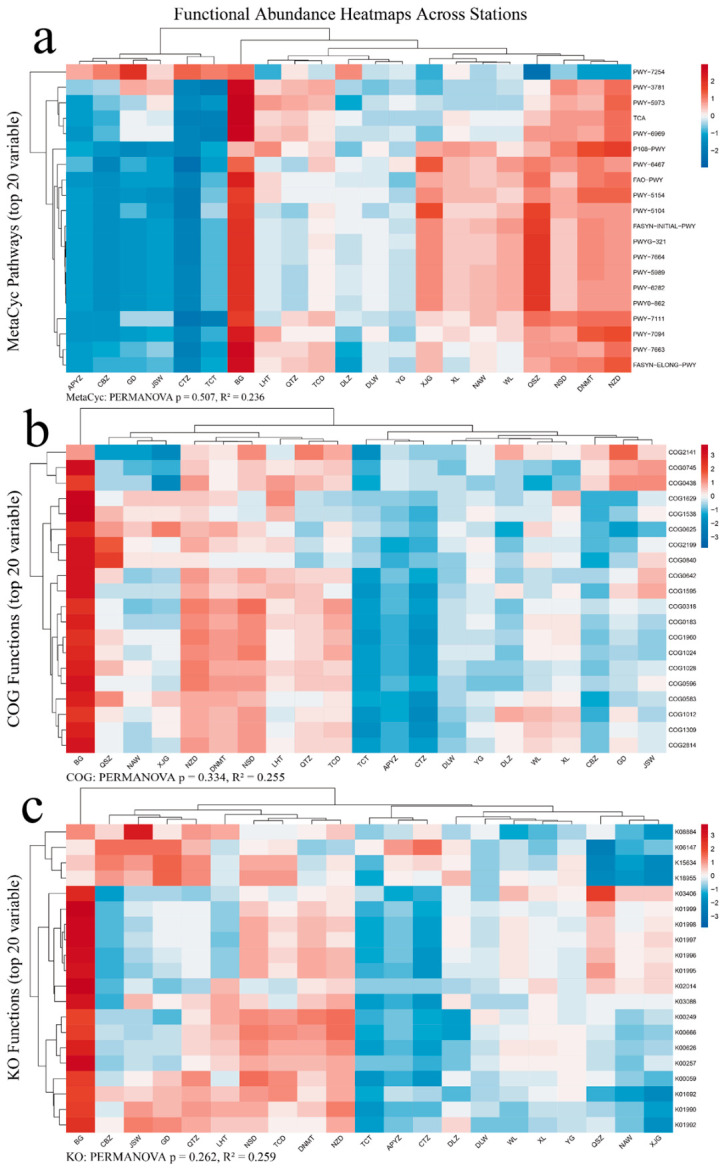
Predicted functional profiles of bacterial communities in the coastal waters of the Leizhou Peninsula, inferred using PICRUSt2 and annotated at three complementary levels: (**a**) MetaCyc pathways (top 20 most variable), (**b**) COG functional categories (top 20 most variable), and (**c**) KEGG Orthology (KO) functions (top 20 most variable).

Given that a substantial proportion of ASVs (34.49%) could not be assigned to specific genera, functional predictions were constrained to broad metabolic categories to avoid over-speculation. PICRUSt2 predictions at KEGG Level 2 indicated that the potential bacterial functional profiles were consistently dominated by core physiology, particularly Carbohydrate, Amino Acid, Lipid, and Energy Metabolism, alongside Membrane Transport (Figure S1). In contrast, pathways related to higher-organism systems remained negligible throughout the dataset. The functional composition exhibited remarkable conservatism across all sampling sites, indicating strong functional redundancy. Despite this overall stability, specific sites subject to anthropogenic pressure exhibited distinct potential metabolic signatures. For example, the BG site adjacent to an iron and steel plant, and some aquaculture zones (such as NZD, DNMT, NSD sites within Zhanjiang Bay, China), displayed elevated relative abundances of these core metabolic pathways compared to other sites (Figure 12). These results suggested that while the overall functional capacity was stable, local anthropogenic nutrient inputs might stimulate an enhanced potential for specific metabolic activities in certain bacterial communities. However, given that zone-level physicochemical variables did not reveal statistically significant gradients between site types, these differences should be interpreted cautiously and may reflect local environmental variability rather than direct anthropogenic forcing.

## 4. Discussion

Unraveling the spatiotemporal distribution of bacterial communities is fundamental to understanding coastal ecosystem function [34]. Over the past decade, numerous studies have investigated bacterial community dynamics in Chinese coastal ecosystems, including the South China Sea [34], Beibu Gulf [35], Yellow River Delta [36], and Pearl River Estuary [37]. These studies have consistently shown that environmental variables such as temperature, salinity, nutrient concentrations, and dissolved oxygen play dominant roles in driving community structure and diversity. However, despite these advances, studies focusing on the tropical coastal waters of the Leizhou Peninsula, a region influenced by complex monsoonal [38], hydrological [39], and anthropogenic processes [25], remain limited. Therefore, this study aims to characterize the seasonal similarities and differences in bacterial community composition and elucidate how environmental factors regulate bacterial diversity and distribution in the coastal waters of the Leizhou Peninsula, China.

### 4.1. Relationship Between Bacterial Community Composition and Diversity in Coastal Waters of the Leizhou Peninsula

The bacterial community in the coastal waters of the Leizhou Peninsula was dominated by *Proteobacteria* (32.4%), *Cyanobacteria* (23.3%), *Actinobacteriota* (20.6%), *Bacteroidota* (13.5%), and *Firmicutes* (2.54%), which is consistent with previous findings from similar subtropical marine environments. Zhu et al. [40] reported that Proteobacteria was the predominant phylum (44.7%) in surface sediments of the South China Sea. Similarly, Gong et al. [35] found that Proteobacteria was the predominant phylum (52.3%), followed by Bacteroidetes (7.73%), in mangrove sediments along the Beibu Gulf, China. In the Xisha Islands of the South China Sea, Wang et al. [41] observed that *Proteobacteria* and *Cyanobacteria* were the two major bacterial groups in marine waters. Furthermore, *Faecalibacterium*, a genus typically considered an essential human gut bacterium and anthropogenic indicator [42], accounted for only a minor component in abundance within the *Firmicutes* phylum in our study. This low relative abundance suggests limited human fecal contamination in the studied environment.

At the genus level, the bacterial community was dominated by a large fraction of unclassified taxa (34.49%), followed by *Cyanobium*_PCC-6307 (12.46%), *Synechococcus*_CC9902 (6.74%), *Candidatus*_*Actinomarina* (5.05%), and HIMB11 (4.99%) (Figure 2). High proportions of unclassified 16S rRNA gene sequence reads are common in marine microbial surveys and likely reflect the inadequate representation of diverse bacterial lineages in reference databases, as well as the prevalence of environmentally adapted clades in subtropical marine habitats such as the South China Sea [40]. *Cyanobium* (*Cyanobium*_PCC-6307) was the single most abundant annotated genus in our dataset and showed a clear warm season peak. Seasonal peaks of *Cyanobium* in subtropical coastal and estuarine waters have been documented previously; for example, Zhang et al. [37] reported that *Cyanobium*_PCC-6307 displayed elevated relative abundances within particle-attached (PA) bacterial assemblages during the summer time, contrasting with the diminished prevalence in these fractions during winter. This is likely because *Cyanobium*_PCC-6307 can form aggregates or blooms in large fractions in the summer when nutrient concentrations and water temperature are higher. Similarly, Wang et al. [41] found *Synechococcus* to be a dominant cyanobacterial genera in oligotrophic reef waters of the Xisha Islands, which was positively correlated with temperature. *Synechococcus*_CC9902 also showed seasonality in our samples and is commonly reported as a dominant phototrophic genus in coastal systems worldwide [43]. The dynamics and seasonal succession of *Synechococcus* clades are well documented in many coastal time series, and are frequently driven by temperature, nutrient availability and water column stratification [44]. These parallels suggest that the summer-time increase in *Cyanobium* in the Leizhou Peninsula likely reflects enhanced photoautotrophic activity under warmer conditions, as well as responses to seasonal nutrient regimes. The presence of *Candidatus*_*Actinomarina* at appreciable relative abundance is consistent with metagenomic and amplicon studies, which have identified this streamlined marine *Actinobacteria* lineage as a widespread and often abundant member of surface ocean bacterioplankton [45]. Members of the “*Ca. Actinomarinales*” are reported from epipelagic waters globally and are interpreted as oligotrophic specialists with small cells and compact genomes [45]. Their detection here may reflect the contribution of true pelagic, low-nutrient adapted lineages mixed into nearshore waters seasonally. The occurrence of HIMB11-type taxa in our samples is consistent with many coastal surveys. For instance, HIMB11 (a Roseobacter related OTU) has been isolated from coastal seawater and is representative of diverse *Alphaproteobacteria* that occupy photoheterotrophic or organotrophic niches in coastal ecosystems. Additionally, members of the Roseobacter clade contribute substantially to heterotrophic processing of organic matter and to sulfur and DOM transformations [46].

High levels of species richness, evenness, and diversity are generally indicative of greater community complexity and enhanced ecological stability [47]. Biodiversity metrics therefore serve as fundamental indicators for evaluating microbial community structure, as microbial diversity is essential for maintaining ecosystem functionality and resilience under fluctuating environmental conditions [48]. Our results demonstrated clear seasonal fluctuations in α-diversity, with indices such as Shannon, Chao1 and ACE showing minimum values in September and peaking in winter. These findings align with observations from other aquatic systems: Yan et al. [49] documented higher bacterial diversity in winter than in summer in a subtropical Chinese coastal region, and Shang et al. [50] similarly reported winter peaks in bacterial richness and diversity in Hulun Lake, northern China.

The strong negative correlation between water temperature and multiple diversity indices observed in our study (Shannon: R = –0.510; Simpson: R = –0.574) further supports the idea that elevated temperature may suppress bacterial diversity in subtropical coastal waters, aligning with findings by Yan et al. [49] and Liu et al. [51]. In contrast, the positive correlations between PO_4_^3−^-P and SiO_3_^2−^-Si with diversity metrics indicate that nutrient availability enhances community richness and evenness. This is consistent with Cui et al. [52], who similarly observed positive relationships between nutrient concentrations and α-diversity in surface marine bacterial communities.

β-diversity analysis based on NMDS ordination showed significant seasonal segregation of bacterial communities (*p* < 0.01). This seasonal clustering pattern closely resembles that observed by Kobiyama et al. [53] in Ofunato Bay, Japan. This long-term metagenomic survey revealed distinct winter–spring and summer–autumn assemblages, strongly structured by environmental gradients including temperature, salinity, and nutrient concentrations. Similarly, in the coastal waters of the Leizhou Peninsula, our results indicated that seasonal transitions in physicochemical parameters—particularly temperature and phosphate availability—drove pronounced shifts in bacterial community composition. These findings indicate that bacterial diversity and community assembly processes in tropical coastal ecosystems are jointly modulated by seasonal environmental forcing and nutrient regimes.

### 4.2. Seasonal Dynamics in Bacterial Community Assembly Mechanisms and Bacterial Co-Occurrence Networks in Coastal Waters of the Leizhou Peninsula

The assembly of bacterial communities is governed by a dynamic interplay of deterministic and stochastic processes that complement and constrain one another across spatial and temporal scales [54]. In our tropical coastal system, the relative influence of these processes exhibited distinct seasonal patterns, consistent with the view that their balance is shaped by environmental context. For example, Yan et al. [55] found taxonomic dependency and spatial heterogeneity in assembly mechanisms in the East China Sea, with abiotic gradients shifting the balance towards determinism. In our study, the bacterial community in Autumn was strongly governed by deterministic processes. This is consistent with findings by Mo et al. [56] along a latitudinal gradient, where strong environmental filtering selected specialist taxa and suppressed stochastic drift. Moreover, Zhou and Ning [57] noted that stable or homogeneous environments often favor stochastic assembly, whereas extreme or highly selective conditions drive deterministic processes. Conversely, the winter season (December) was characterized by a dominance of stochastic processes, as indicated by the highest neutral model fit (R^2^ = 0.626) and broader niche breadths. This suggests that the tropical winter in the Leizhou Peninsula presents a relatively homogeneous environment with reduced selective pressure, where generalist taxa with broad environmental tolerances can disperse freely, allowing ecological drift and random dispersal to become the primary drivers of community assembly [58]. This pattern contrasts with temperate systems such as the Western English Channel, where winter imposes harsh selective filters [19].

Co-occurrence network analysis revealed a distinct seasonal reshaping of bacterial interaction topology in the coastal waters of the Leizhou Peninsula, China. Across all four seasons, most pairwise associations were positive, suggesting that cooperative interactions and shared niche preferences dominated over antagonistic relationships—a pattern consistently observed in long-term marine coastal observatories and global ocean datasets [59].

The spring network exhibited the largest size and highest connectivity, suggesting a highly interconnected assemblage during early spring. This aligns with observations of heightened microbial interconnectivity in nutrient-dynamic surface waters, where enhanced metabolic linkages and resource exchange among taxa are thought to dominate [60]. In contrast, the summer network was smaller in node and edge counts but showed higher clustering and density, reflecting tighter local clustering of functionally linked taxa under summer stratification or localized nutrient regimes [59]. In autumn, the network structure underwent a marked shift. While the average degree remained comparable to other seasons, modularity reached its annual maximum (0.437) (Table 2). This is consistent with our finding that deterministic processes dominate in autumn. By winter, the network was sparser and exhibited lower modularity (0.341) compared to autumn. This topological simplification indicates a decline in community compartmentalization. In contrast to temperate systems such as Lake Taihu, where strong environmental filtering in winter drives complex, highly modular networks [61], our results suggest that the tropical winter in the Leizhou Peninsula is dominated by stochastic processes. Topological role analysis further revealed an absence of clear Module Hubs and Network Hubs, suggesting that no single taxon consistently served as a dominant structural keystone. Furthermore, connectors were present only sporadically and showed a seasonal decline, suggesting that putative keystone roles were transient and season specific. This pattern consistent with recent cautions that keystone taxa inferred from co-occurrence networks require complementary experimental validation [62].

While these networks reflect statistical covariation rather than direct biological interactions, the observed seasonal shifts provide robust evidence of changing community organization—from high connectivity during spring reassembly to stronger niche partitioning in winter. We further recommend follow-up analyses to disentangle environment-driven covariation from putative biotic interactions.

### 4.3. Functional Prediction of Bacterial Communities in Coastal Waters of the Leizhou Peninsula

Predictive functional profiling based on 16S rRNA gene sequences is increasingly employed to infer the metabolic responses of microbial assemblages under varying environmental conditions [63,64]. Across all sites, the relative abundances of functional genes involved in Carbohydrate Metabolism, Amino Acid Metabolism, and Energy Metabolism were significantly higher than those of other categories, indicating that these metabolic processes are fundamental for bacterial communities in the Leizhou Peninsula, China. Our results indicated that seasonal variations had a negligible impact on the overall functional structure of bacterial communities. This finding contrasts with the strong seasonal functional patterns observed by Ward et al. [65] in a temperate estuary. This inconsistency may be attributed to climatic differences between the two study regions. Subtle differences in predicted metabolic potential were observed among sites, with stations BG, NZD, DNMT, and NSD showing marginally elevated relative abundances of certain functional categories compared to less impacted sites such as TCT, APYZ, and CTZ. However, given that zone-level physicochemical variables did not reveal statistically significant gradients between site types, these spatial differences in predicted functional potential should be interpreted with caution. All functional profiles presented here represent inferred metabolic potential derived from 16S rRNA amplicon data rather than direct measurements of gene expression or metabolic activity, and conclusions regarding actual functional responses to anthropogenic pressures require validation through metatranscriptomic or metagenomic approaches.

### 4.4. Environmental Filtering and Seasonal Drivers of Bacterial Community in Coastal Waters of the Leizhou Peninsula

RDA identified salinity as the key driver of bacterial community composition (*p* < 0.001), with particularly strong effects in summer and winter. This finding supports the recognition of salinity as a global stressor in coastal transition zones, where it acts as a primary biogeographical barrier between freshwater and marine lineages [66]. Salinity imposes significant osmotic pressure on microbial cells, strongly selecting taxa with specific osmoregulation capabilities—a factor that can suppress microbial network interactions and reduce community diversity under high salinity condition [67]. Furthermore, previous studies in estuarine sediments have confirmed that bacterial abundance is significantly correlated with salinity gradients, reinforcing its role as a deterministic filter [68].

While salinity establishes the baseline, the significant influence of COD and DO in our study highlights the critical role of anthropogenic organic enrichment in further shaping community structure. Although salinity typically governs broad distribution patterns, high nutrient inputs can uncouple bacterial activity from salinity gradients, stimulating elevated metabolic rates and reshaping community composition even in saline waters [69]. This pattern is consistent with observations in other anthropogenically influenced estuaries, where organic matter and nutrient loading serve as important secondary drivers, selecting for bacterial groups capable of degrading complex organics [70]. Additionally, the correlation with DO may reflect the metabolic demand of these communities [71].

The temporal shifts in key drivers—from salinity (in summer or winter) to COD and pH (during seasonal transitions)—reflect the dynamic hydrology of the Leizhou Peninsula. Similar spatial shifts have been documented in the Pearl River Estuary, where environmental factors such as organic carbon and salinity differentially shape bacterial sub-communities [72]. For instance, seasonal variations in freshwater runoff and precipitation can modify the extent of saltwater intrusion, salinity acting as a dominant filter during dry season, while other factors become more influential in the wet season [67]. Moreover, physical factors such as temperature and depth—which showed limited effects in our study—typically interact with these chemical drivers to modulate community assembly processes during specific seasonal windows, such as summer stratification [73].

### 4.5. Limitations and Summary

Notwithstanding these insights, several limitations in this study should be acknowledged. First, functional profiles were inferred using PICRUSt2 based on 16S rRNA amplicons. While informative, these predictions represent potential metabolic capabilities rather than active gene expression; future studies employing metagenomics or metatranscriptomics are recommended to validate the active metabolic pathways involved in specific biogeochemical cycles. Second, this study focused exclusively on bacterioplankton in the water column. Given the dynamic exchange between the water column and benthos in shallow coastal ecosystems, future research should simultaneously investigate sediment microbiomes to obtain a holistic understanding of the benthic–pelagic coupling in the Leizhou Peninsula, China.

## 5. Conclusions

In this study, we investigated the diversity, composition, and seasonal and spatial variation in bacterial communities in the coastal waters of the Leizhou Peninsula using high-throughput sequencing. Our results demonstrate that seasonal environmental heterogeneity is the primary driver shaping bacterial community structure and diversity. Alpha diversity exhibited significant seasonal variation, while temperature and phosphate availability acted as key regulators based on Spearman correlation analysis. Salinity was identified as the dominant deterministic factor governing community composition, explaining 20.7–37.9% of compositional variation across seasons, as determined by RDA. We also revealed a distinct seasonal succession in community assembly mechanisms, shifting from deterministic filtering in autumn to stochastic processes in winter—a transition further supported by network topology. Functional predictions indicated that while core metabolic potential remained stable, subtle spatial differences in predicted functional profiles were observed across site types, though causal attribution to anthropogenic forcing requires further metagenomic validation. These findings provide a seasonal mechanistic framework for understanding how environmental filtering, stochastic dispersal, and hydrodynamic forcing collectively shape bacterial communities structure in tropical–coastal ecosystems, with implications for predicting bacterial community responses to ongoing environmental change.

## Figures and Tables

**Table 1:** Contributions of environmental factors to bacterial community variations based on redundancy analysis (RDA).

Basin	Explained Variation (%)			Explanatory Variables (Contribution %)
	Axis 1	Axis 2	All Axes	
Spring	15.45	10.79	26.24	COD (14.7), Depth (14.1), T (20.7) Salt (37.9), DO (18.9), pH (13.9) Salt (20.7), DO (13.2), T (14.6), pH (17.2) Salt (35.2)
Summer	40.02	15.50	55.52	
Autumn	19.42	13.13	32.55	
Winter	46.34	11.01	57.35	

**Table 2:** Topological parameters of the seasonal bacterial co-occurrence networks, including number of nodes, edges, average degree, average path length, density, and modularity.

Basin	Node	Edge	Average Degree	Average Path Length	Network Diameter	Clustering Coefficient	Density	Modularity	Statistical Inference
Spring	163	2228	27.337	2.321	7	0.608	0.169	0.306	4691.327
Summer	122	1259	20.639	2.317	5	0.599	0.171	0.272	2521.706
Autumn	128	1413	22.078	2.413	8	0.678	0.174	0.437	3222.525
Winter	149	1634	21.933	2.468	6	0.621	0.148	0.341	3320.361

## Data Availability

Datasets utilized for this research are available from the Sequence Read Archive (SRA) under BioProject PRJNA1453757.

## Supplementary Materials

The following supporting information can be downloaded at: https://www.mdpi.com/article/10.3390/microorganisms14061359/s1, Figure S1: Predicted functional profiles of bacterial communities in the coastal waters of the Leizhou Peninsula, inferred using PICRUSt2 and summarized at KEGG Pathway Level 2; Figure S2: Heatmap of environmental variables across all sampling stations and seasons in the coastal waters of the Leizhou Peninsula, China. Rows represent environmental parameters (Depth, T, pH, Salinity, Chl-a, DIN, SiO_3_-Si, PO_4_-P, and COD) with value ranges indicated in parentheses. Columns represent individual samples grouped by zone (Industrial zone, Tourist area, and Aquaculture zone) and month (December 2022, September 2022, June 2023, and March 2023). Color intensity reflects relative magnitude from minimum (light purple) to maximum (dark purple) within each variable; Table S1: Statistics of obtained sequence bacterial 16S rRNA gene amplicon pyrosequencing.
