# Supplementary material for: Spatiotemporal Dynamics and Assembly Mechanisms of Bacterial Communities in Tropical-Subtropical Coastal Waters of the Leizhou Peninsula, China
Source: Microorganisms. 2026 Jun 17;14(6):1359. doi: 10.3390/microorganisms14061359 (PMC13304073; doi:10.3390/microorganisms14061359)
Supplement: Supplementary file 1 [file microorganisms-14-01359-s001.zip › Wei et al., supplementary Figure 20260601.pdf]

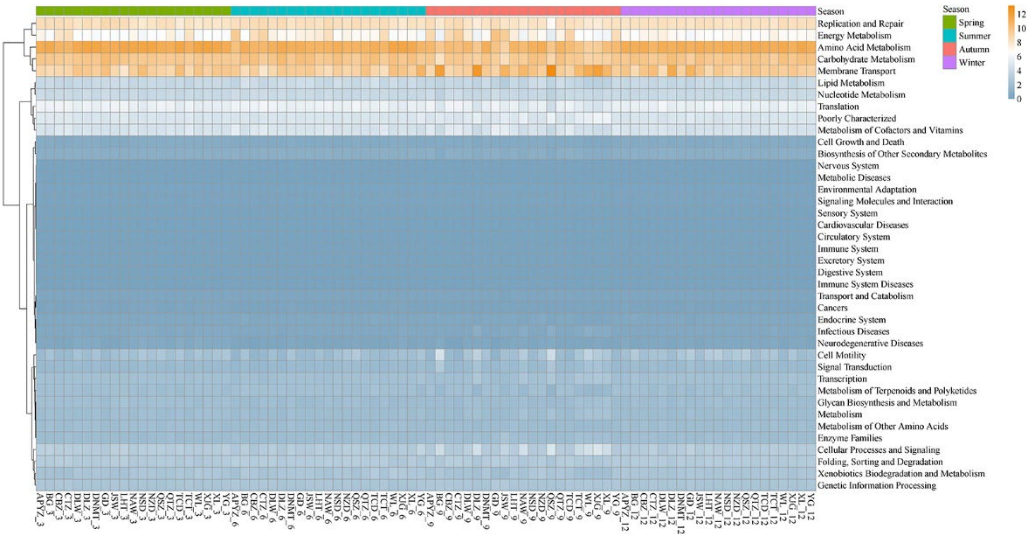

Figure S1. Predicted functional profiles of bacterial communities in the coastal waters of the Leizhou Peninsula, inferred using PICRUSt2 and summarized at KEGG Pathway Level 2.

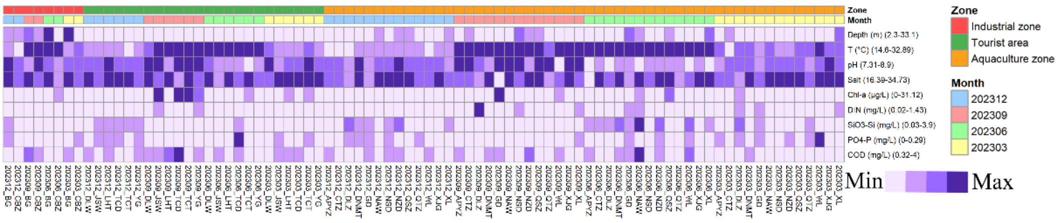

Figure S2. Heatmap of environmental variables across all sampling stations and seasons in the coastal waters of the Leizhou Peninsula, China. Rows represent environmental parameters (Depth, T, pH, Salinity, Chl-a, DIN, SiO_3-Si, PO_4-P, and COD) with value ranges indicated in parentheses. Columns represent individual samples grouped by zone (Industrial zone, Tourist area, and Aquaculture zone) and month (December 2022, September 2022, June 2023, and March 2023). Color intensity reflects relative magnitude from minimum (light purple) to maximum (dark purple) within each variable.
